# Permittivity-Inspired Microwave Resonator-Based Biosensor Based on Integrated Passive Device Technology for Glucose Identification

**DOI:** 10.3390/bios11120508

**Published:** 2021-12-09

**Authors:** Wei Yue, Eun-Seong Kim, Bao-Hua Zhu, Jian Chen, Jun-Ge Liang, Nam-Young Kim

**Affiliations:** 1 Radio Frequency Integrated Circuit (RFIC), Kwangwoon University, Kwangwoon-ro, Nowon-gu, Seoul 01897, Korea; yuewei@kw.ac.kr (W.Y.); esk@kw.ac.kr (E.-S.K.); zhuwangwhy@kw.ac.kr (B.-H.Z.); cnjacob@kw.ac.kr (J.C.); 2 Engineering Research Center of IoT Technology Applications (Ministry of Education), Department of Electronic Engineering, Jiangnan University, Wuxi 214122, China

**Keywords:** integrated passive device (IPD), glucose identification, microwave, biosensor, permittivity, air-bridge capacitor

## Abstract

In this study, we propose a high-performance resonator-based biosensor for mediator-free glucose identification. The biosensor is characterized by an air-bridge capacitor and fabricated via integrated passive device technology on gallium arsenide (GaAs) substrate. The exterior design of the structure is a spiral inductor with the air-bridge providing a sensitive surface, whereas the internal capacitor improves indicator performance. The sensing relies on repolarization and rearrangement of surface molecules, which are excited by the dropped sample at the microcosmic level, and the resonance performance variation corresponds to the difference in glucose concentration at the macroscopic level. The air-bridge capacitor in the modeled RLC circuit serves as a bio-recognition element to glucose concentration ($\varepsilon_{glucose}C_{0}$), generating resonant frequency shifts at 0.874 GHz and 1.244 GHz for concentrations of 25 mg/dL and 300 mg/dL compared to DI water, respectively. The proposed biosensor exhibits excellent sensitivity at 1.38 MHz per mg/dL with a wide detection range for glucose concentrations of 25–300 mg/dL and a low detection limit of 24.59 mg/dL. Additionally, the frequency shift and concentration are highly linear with a coefficient of determination of 0.98823. The response time is less than 3 s. We performed multiple experiments to verify that the surface morphology reveals no deterioration and chemical binding, thus validating the reusability and reliability of the proposed biosensor.

## 1. Introduction

Owing to changing lifestyles and the prevalence of obesity worldwide, diabetes in people is increasing at an alarming rate [1]. It is the leading global cause of death resulting from severe complications, including heart attack, stroke, and kidney failure [2]. Therefore, accurate and early detection of glucose levels is essential for timely diagnosis of diabetes in overweight people and diabetic patients to monitor their health. Typically, fasting plasma glucose (FPG) standard with a blood glucose level of 70 mg/dL (3.9 mM) and 100 mg/dL (5.6 mM) are considered acceptable for glucose biosensor detection [3].

Existing studies have developed glucose biosensors based on various detection technologies, such as optical, electrochemical, and microwave-based techniques [4,5]. Optical techniques analyze the optical properties and multiple responses of the sample under test (SUT) to light [6] based on selective absorption and rotatory dispersion [7]. Although optical technology is fast and non-consumable, it requires expensive analytical equipment [6]. Conversely, electrochemical methods generally exploit specific reactions or binding with SUT that occur on the surface of the distinctively modified electrodes [8,9,10]. Thus, electrochemical technology achieves high accuracy and sensitivity despite the high cost of consumable reagents and a low tolerance for environmental changes. By contrast, microwave-based technology relies on particular electromagnetic properties of SUT and offers higher stability and improved resistance to disturbances. Moreover, as it is more cost-effective than the aforementioned technologies with the potential to be non-invasive, it has been developed for glucose detection [11,12,13,14]. 

Most microwave biosensors adapt the resonator structure and model it as an RLC circuit, wherein the capacitor is used for bio-recognition [11]. The sensing is based on the capacitive effect derived from the interaction between SUT and electromagnetic wave and is limited by intrinsic resonator performance and reaction intensity [15]. However, the resonator is expected to have narrowband performance and a sensitive surface determined by designs, structure, and materials [15], whereas the reaction intensity is negatively correlated with the resonant frequency [16]. Most sensors use planar configurations that are optimized by designs. Sharafadinzadeh et al. reported a planar glucose biosensor based on a split ring resonator (SRR), which exhibited a 0.0008 MHz frequency shift per mg/dL glucose variation [17]. Liu et al. reported a planar biosensor based on a hairpin resonator for biological cell detection, reflecting each cell number in S11 magnitude at 0.83 dB variation [18]. Govind and Akhtar proposed a planar glucose biosensor based on SRR, incorporating the interdigital capacitor (IDC) for enhanced capacitive effect. The sensors reflected the concentration in resonant frequency variation at 0.026 MHz per mg/dL, responding to concentrations ranging from 0 to 5000 mg/dL [19]. Manik and Muhannad proposed a glucose-responsive hydrogel-interlayer radio-frequency (RF) resonator with sensitivity of 0.333 MHz per mg/dL [20]. In comparison with the planar resonator, previously proposed integrated passive device (IPD) resonators with multiple layers fabricated on a low-loss gallium arsenide (GaAs) substrate exhibited complicated fabrication with higher performance by improving the substrate materials, fabrication, and structure [14,15,16]. However, further optimization is required for improving the sensing performance and analysis of previously reported sensors at the microcosmic and macroscopic levels when applied to glucose detection [21].

Therefore, we propose a glucose resonator-based biosensor with improved sensing performance in this study, using IPD fabrication technology and improved sensing principal analysis. The biosensor fabricated on a GaAs substrate comprises a circle-shaped spiral inductor and air-bridge capacitors on the exterior structure. The internal capacitor provides a sensitive surface and clear indicator. Furthermore, after dropping the SUT on the sensing section, the microcosmic variations in surface molecules and macroscopic responses are analyzed. The former analysis depends on two kinds of effect: the introduction of glucose and water molecules affects the electromagnetic field distribution, and the existing electromagnetic wave contributes to the polarization of the molecules. The overall transmission characterization is changed by the combined effect, reflected in S-parameter changes. The latter analysis utilized the air-bridge capacitor as the bio-recognition element to SUT permittivity, responding proportionally to $\varepsilon_{glucose}C_{0}$ and resulting in a substantially higher sensitivity of 1.38 MHz per mg/dL than described in existing reports. Moreover, we investigated the reusability and repeatability through multiple experiments of surface morphology to validate the high performance of the biosensor.

## 2. Materials and Methods

### 2.1. Design and Optimization

**Figure 1:**
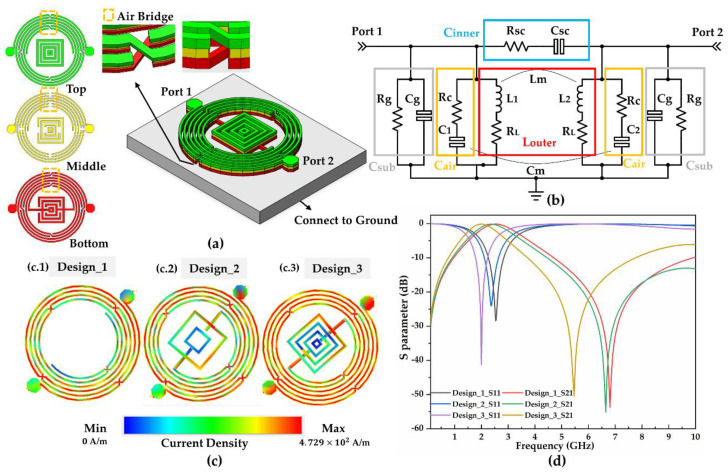
The conception of the proposed resonator-based biosensor: (**a**) The layout of the proposed biosensor with the three-layered structure; (**b**) The equivalent circuit of the proposed biosensor; (**c**) Various internal designs with current distribution conditions at the resonant frequency: (**c.1**) Current density distribution of Design.1; (**c.2**) Current density dis-tribution of Design.2; (**c.3**) Current density distribution of Design.3; (**d**) Simulated S-parameters of the designs.

The proposed glucose microwave biosensor was developed on the basis of a resonator, wherein the resonant frequency was determined by the total inductance ($L_{total}$) and capacitance ($C_{total}$), as indicated in Equation (1) [22]. In this design, $L_{total}$ and $C_{total}$ are primarily derived from the exterior design, which includes the circle-shaped spiral inductance and five air-bridge capacitors, as depicted in Figure 1a. Furthermore, the inner square-shaped spiral capacitor ($C_{inner}$) contributes to the total capacitance ($C_{total}$) and adjusts resonance conditions for improved sensing performance. Additionally, multiple layers increase the mutual coupling effect, further enhancing the total inductance and capacitance and generating stronger electromagnetic intensity.

(1) $$f_{c}=\frac{1}{2\pi \sqrt{L_{total}C_{total}}}$$

Figure 1b illustrates the equivalent circuit of the proposed biosensor composed of the exterior structure ($L_{outer}$, $C_{air}$), the internal structure $(C_{sc}$), and substrate structure ($C_{sub}$). Herein, $C_{g}$ and $R_{g}$ denote the coupling capacitance and leakage resistance, respectively, of the GaAs substrate ($C_{sub}$). The exterior design comprises the circle-shaped spiral inductor ($L_{outer}$) and air-bridge capacitor ($C_{air}$) that provide high inductance and capacitance with compact size, generating a strong electromagnetic field for a sensitive response. In the $L_{outer}$ section (Figure 1b), $L_{m}$ and $C_{m}$ denote the mutual inductor coupling and capacitor coupling effect, respectively. The inductance of the single-layer inductor is modeled approximately as $L_{1,2}$ based on Equation (2) [23]:

(2) $$\;L_{1,2}=\frac{\mu_{0}n^{2}d_{avg}c_{1}}{2}\left[\ln\left(\frac{c_{2}}{\rho }\right)+c_{3}\rho +c_{4}\rho^{2}\right]$$

where $c_{1}$, $c_{2}$, $c_{3}$, and $c_{4}$ indicate the constant coefficients of the shape of the layout [24]. Table 1 summarizes the coefficients of various shapes, such as square, hexagonal, octagonal, and circular layouts; we chose the circle shape for its highest attribution to the overall inductance [24,25]. Furthermore, n and $d_{avg}$ in Equation (2) denote the number of turns and average diameter of the circular inductor, respectively; and ρ represents the fill ratio, which can be expressed using Equation (3) as:

(3) ρ=(dout−din)/(dout+din)

In this design, the multilayer inductor-capacitor (L-C) resonator consists of the bottom, middle, and top layers. Spiral metal lines are 15 µm in width, the gap is 15 µm, and the turns on each side number 4 and 6, respectively. Furthermore, the resonator is attached to a sub-board PCB to assist with the impedance match and common ground.

The air-bridge capacitors corresponding to $C_{air}$ are introduced via multiple layers with low parasitic inductance and high parasitic capacitance. In the red rectangles of the air-bridge capacitor area in Figure 1a, the middle layer provides the capacitor gap with a size of 45 µm × 30 µm × 1.8 µm. The bottom and top layers were designed as the capacitor plates by crossed connection with 15 µm width, offering a plate area of 225 µm^2^ of capacitors sensitive to the environment. In Figure 1b, $R_{c}$ denotes the resistance effect, and the capacitance of the capacitor was modeled approximately as $C_{1,2}$, calculated using Equation (4) [26]:

(4) $$C_{1,2}=\frac{S\varepsilon_{0}\varepsilon_{r\;}}{d}=\varepsilon_{r}C_{0}$$

where $\varepsilon_{0}$ denotes vacuum permittivity, $\varepsilon_{r}$ indicates the dielectric constant of interplate filling material, and $C_{0}$ represents the capacitance of intermediate vacuum medium per unit area. 

Based on the aforementioned primary exterior design (Figure 1(c.1)), the electrical field distribution was simulated by the Advanced Design System (ADS) 2016.0 simulator (Keysight Technologies). Reaction densities of moderately strong current density at the surface and high current density at the air-bridge capacitor area were generated. Moreover, the indicator of a resonance condition at 2.5 GHz/−28 dB and full width at half maximum (FWHM) of 0.7 GHz were observed. Higher current density and peaks with lower FWHM and higher absolute value contribute to reaction density and indicator clarity. Therefore, we constructed a tunable internal capacitor structure with intense surface current density and enhanced indicator performance, which is represented as $C_{inner}$ in Figure 1b. The capacitance$\;(C_{sc}$) was calculated using Equation (5) [23]:

(5) $$C_{sc}=\left[\varepsilon_{0}\left(\frac{1+\varepsilon_{s}}{2}\right)\frac{K\left(\sqrt{1+k^{2}}\right)}{K\left(k\right)}+\varepsilon_{0}\frac{t}{a}\right]L_{c}$$

where $\varepsilon_{0}$ and $\varepsilon_{s}$ denote the permittivity of free space and GaAs substrate, respectively, $L_{C}$ indicates the total length of the spiral capacitor line, and K(k) represents the first type of elliptic integral. 

Figure 1c depicts the current distribution density of the exterior design (Design_1) and those with various central capacitors (Design_2 and Design_3) at the resonant frequency. Design_3 significantly promotes the current density of the external spiral inductor, generating a denser current surface with enhanced reaction density. Figure 1d illustrates the S-parameter simulation of the aforementioned designs and indicator performance. As indicated in the figure, Design_3 with a resonant frequency of 2 GHz exhibits more potential in sensing applications than Design_1 and Design_2, resulting from a lower FWHM of 0.5 GHz and a higher magnitude value of −42 dB. Consequently, the glucose biosensor was fabricated using Design_3, which was selected considering its stronger reaction density and higher indicator clarity in comparison with other designs.

### 2.2. Fabrication Process

**Figure 2:**
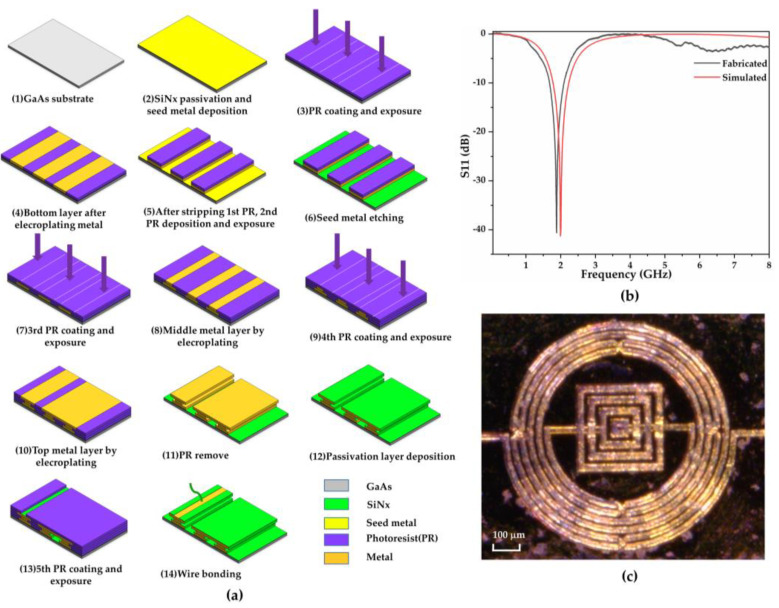
Fabrication process and results for the sensors: (**a**) The fabrication process; (**b**) Frequency response of the simu-lation and fabrication; (**c**) A microscopic picture of the fabricated biosensor.

Figure 2a depicts the fabrication process for the device, which was divided into three metal layers based on bottom-up IPD fabrication. We selected GaAs substrate as a semi-insulating material to reduce the parasitic effect significantly and increase the resonant frequency and quality of high frequency [27]. 

Step 1: Before the fabrication process, the GaAs substrate (200.1 μm) was pre-washed with acetone and deionized (DI) water to obtain a smoother surface with stronger layer adhesion. Steps 2–6: Initially, the silicon nitride (SiNx, 200 nm) passivation layer was deposited on the substrate through a plasma-enhanced chemical vapor deposition process, providing strong adhesion between the metal layer and GaAs wafer. After the seed metal layer of Ti/Au (20/80 nm) was deposited by sputtering, the first negative photoresistor (PR) was spin-coated to define the pattern of the first metal layer. Subsequently, the first Cu/Au (4.5/0.5 μm) metal layer was fabricated through electroplating. After the first PR layer was stripped by acetone solution and a lift-off machine, the second PR layer was coated and selectively exposed for the corresponding pattern with the first metal layer protecting it. Furthermore, the first seed metal layer was removed selectively.

Steps 7 and 8: A third negative PR was applied, followed by an exposed mask to define the second metal layer pattern. This defines the gap of the air-bridge capacitor. Subsequently, the second Cu (1.8 μm) metal layer was electroplated on the surface corresponding to the PR pattern. Steps 9–11: Another negative PR was coated, which was followed by a selectively exposed mask for the third metal layer pattern. Subsequently, a third metal layer (Cu/Au) with a thickness of 4.5/0.5 μm was sputtered to form the top of the air bridge. The PR layers were removed, and the air-bridge structure was retained. Steps 12–14: The final passivation layer was deposited to protect against any environmental impurities. Another PR was adopted with a particular pattern to generate an open area for inductively coupled plasma dry etching. After etching and PR removal, Au wire bonding was used for the interconnection between the chip ports and the 50 Ω impedance matching line, which was fabricated on the printed circuit board and processed for measurement, enabling its microwave performance. A bottom iron cube was connected to the multilayer structure through central via holes on the backside of the PCB board and worked as additive ground (Figure S1). Furthermore, a small outline transistor package was adopted to protect the Au wire and prevent device damage during the experiment.

As depicted in Figure 2b, minor deviations were observed during the fabrication process, unlike the simulation results, with a frequency shift of −0.20 GHz and S11 deviation of 1 dB. Figure 2c illustrates a microscopic image of the fabricated biosensor.

### 2.3. Preparation of Sample and Apparatus

For the experiment, glucose powder, fructose powder, and glucose oxidase (GOx) were purchased from Sigma-Aldrich (St. Louis, MO, USA), and fetal bovine serum (FBS) was purchased from Welgene and stored at less than −20 °C. All chemicals were used without further purification. The surface morphology of the developed biosensor was characterized using an atomic force microscope (AFM, FC-AM30). The S-parameter measurements were obtained using a vector network analyzer (VNA, 8719ES), measuring the frequency range from 0.05 GHz to 8 GHz in 0.005 GHz steps. Microscopic images were acquired using a microscope (Dino-Lite, AM-413T). 

The concentration of the glucose solution was based on the glucose level of healthy people, ranging from 70 to 125 mg/dL of FPG standard [5]; both hypoglycemia and hyperglycemia ranges were considered. Therefore, samples were prepared in the range from 25 mg/dL to 300 mg/dL in 25 mg/dL increments by dissolving the glucose powder in DI water. All samples were prepared at 26 °C and stored at 0–4 °C.

## 3. Results and Discussion

### 3.1. Characterization and Experimental Process

**Figure 3:**
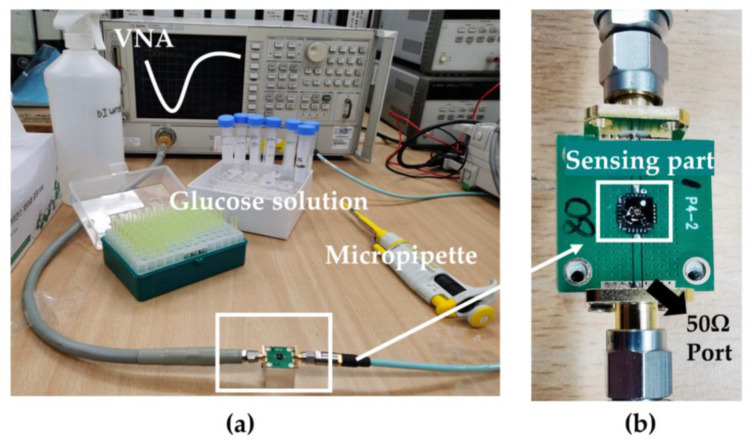
Experimental setup for the tests. (**a**) The experimental setup and preparation for the meas-urement of glucose level. (**b**) The tested device connected to a vector network analyzer (VNA) through a 50 Ω port.

Figure 3 depicts the device, packed with a 50 Ω port for impedance match, connected to the VNA. Before glucose solution measurement, initially, the glucose level identification experiments recorded the bare chip and DI water droplet S-parameters as a reference. Subsequent tests were performed after washing and drying the surface. After several tests in groups, the sensor surface was rinsed with phosphate buffered saline (PBS) and water and dried through a nitrogen drying process. This was followed by another measurement of bare chip S-parameters.

Moreover, to further demonstrate the potential for clinical applications of the sensor, GOx was added because of its specific reaction to glucose (Figure S5) [28]. After measuring the responses to 5 μL 100 mg/dL glucose and fructose serum solution as the control group, mixed 3 μL glucose oxidase solution (no catalyst, 16.80 units), and 2 μL 100 mg/dL glucose and fructose serum solution, the reaction time was set at 10 min.

### 3.2. Reusability and Repeatability

**Figure 4:**
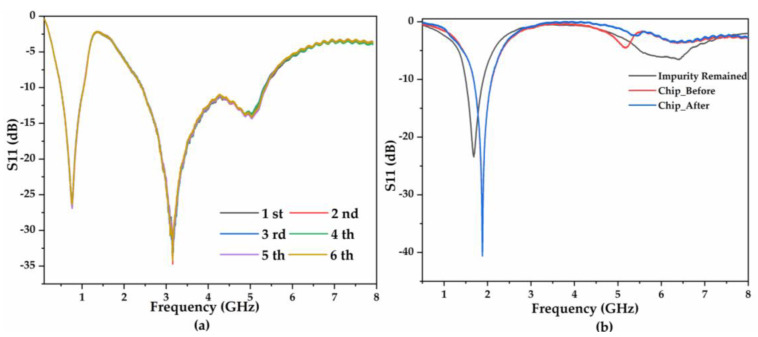
Pre-reliability and reusability investigation. (**a**) Six experiments for testing the 150 mg/dL glucose solution. (**b**) Unloaded chip resonance performance before and after the experiment.

Before performing glucose identification experiments, we investigated the reliability of the biosensor based on the consistency of performance derived from multiple experiments with the 150 mg/dL glucose solution at the macroscopic level (Figure 4a). Periodic detection was performed 6 times, and the observed high consistency verified the repeatability of the sensor for glucose identification with a relative standard deviation (RSD) of 0.11%. Moreover, the reusability was determined by comparing the resonance performance of the unloaded chip before and after the tests, as illustrated in Figure 4b. When the droplet residue or impurity remains on the surface, the center frequency shifts by approximately 0.1 GHz from the unloaded condition and returns to the unloaded condition after being rinsed with PBS/DI water. Typically, the results for the same concentration and the unloaded performance are highly consistent, validating that no apparent frequency deterioration or shift occurs in the entire process. Thus, the sensor can be considered highly reliable and reusable.

**Figure 5:**
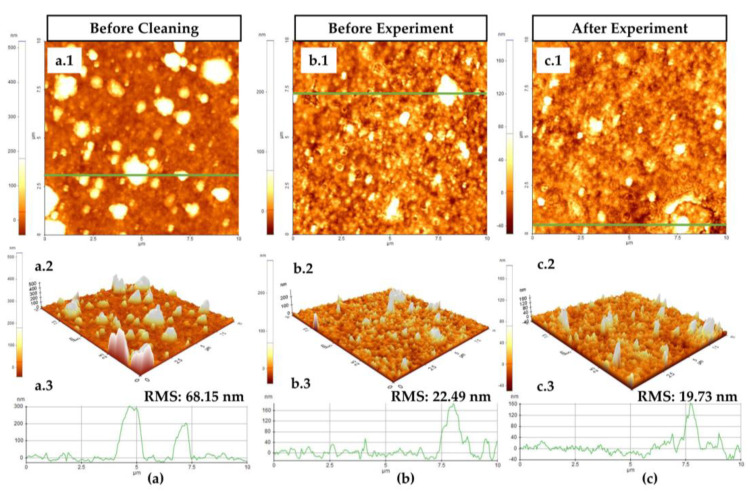
Surface morphological conditions. (**a**) Before cleaning: (**a.1**) the two-dimensional (2-D) view of the unloaded surface; (**a.2**) the three-dimensional (3-D) surface profile; (**a.3**) the line profile for surface roughness distribution of green line. (**b**) Unloaded chip condition before the experiment: (**b.1**) the 2-D view of the surface condition; (**b.2**) the 3-D surface profile; (**b.3**) the line profile distribution of green line; (**c**) Chip condition after multiple experiments: (**c.1**) the 2-D view of the surface condition; (**c.2**) the 3-D surface profile; (**c.3**) the line profile for surface roughness distribution of green line.

To further facilitate reusability and reliability at the microcosmic level, we measured and compared the surface morphology maps before and after the experiment. The root mean square (RMS) value represents the average profile height and indicates the surface roughness level and damaged condition in various scenarios. Figure 5 depicts the characteristics of three states of the chip surface, namely (a) the unloaded chip before cleaning, (b) the unloaded chip surface before the experiment, and (c) the unloaded chip surface after repeating the experiment more than 15 times. 

Figure 5(a.1,a.2,a.3) illustrate the unloaded unpackaged biosensor before cleaning, wherein the RMS value of 68.15 nm is presented in two-dimensional (2-D) and three-dimensional (3-D) representations. Following washing, we observed that the RMS value decreased to 22.49 nm before the experiment, as depicted in Figure 5(b.2). The apparent change implies that the invisible impurities that existed during storage were removed, verifying that cleaning before the test was necessary. As the chip with the droplet could not be tested by the AFM equipment because of its probe distance limitation, we considered the same unloaded biosensor after the experimental process with an RMS value of 19.73 nm (Figure 5(c.3)). The RMS values of the chip before and after the experiment exhibited only a minor deviation. Moreover, the surface profile results indicated high consistency between the groups before and after the experiment, as depicted in Figure 5(c.1,c.2,c.3). The obtained results verified that no chemical immobilization or deterioration on the surface occurred and no frequency shift was generated during the experiment. Thus, the high reusability and reliability of the sensor at both microcosmic and macrocosmic levels could be validated.

### 3.3. Sensing Response Analysis

**Figure 6:**
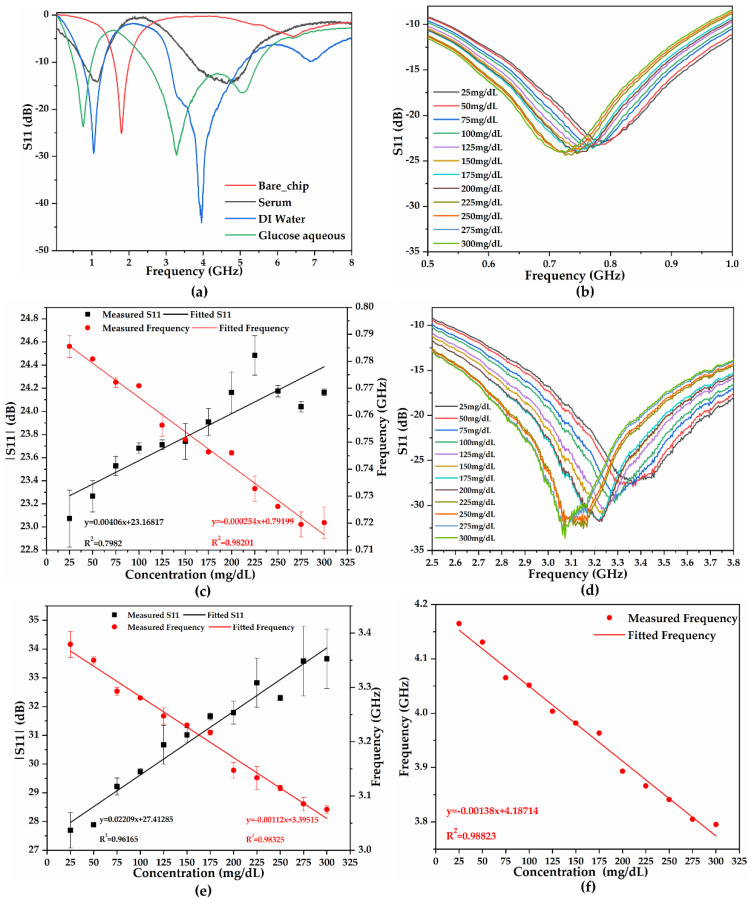
Microwave response under various conditions: (**a**) DI water, serum, and 100 mg/dL glucose aqueous responses compared with the unloaded chip; (**b**) resonant frequency responses of various concentrations at peak 0.8 GHz; (**c**) the linear analysis at 0.8 GHz peak of multiple concentrations with error bars; (**d**) the peak response at 3.2 GHz to multiple concentrations; (**e**) the linear analysis at 3.2 GHz peak of multiple concentrations with error bars; (**f**) summation of the relationship between two peak conditions at various concentrations.

We selected S11 as the reference signal owing to its high resistance to environmental interference in multiple electromagnetic transmission parameters. To evaluate the S11 response of the biosensor to the introduced liquid, DI water was used as a reference and compared with the unloaded condition depicted in Figure 6a. Additionally, serum and 100 mg/dL glucose aqueous solution were added and compared, inducing various responses from the sensor. The bare biosensor resonant frequency at 1.80 GHz shifted to two peaks of 1.054 GHz/−29.354 dB and 3.958 GHz/−44.089 dB for DI water. Herein, the former was considered the decrease from the original frequency of 1.80 GHz because of the air-bridge capacitive effect of the sample; the latter was evaluated as the introduced sample capacitance-like effect (Figure S2). Moreover, no overlap occurred between the responses to different concentrations at the resonant frequency in Figure 6b,d, validating its high sensitivity to support subsequent analysis.

Figure 6b,c illustrate the frequency of approximately 0.8 GHz for peak 1, wherein both amplitude and center frequency differ from linear differential relativity based on various glucose concentrations. The S-parameters were measured three times for each concentration sample to reduce the error, which resulted in a highly consistent standard deviation. The frequency exhibited a linear downtrend from 0.786 GHz to 0.720 GHz as glucose levels increased from 25 mg/ dL to 300 mg/ dL with a fitting curve coefficient of determination (COD) of 0.9820 and a maximum RSD of 0.81% at 300 mg/dL. Additionally, the absolute value of its amplitude increased linearly from 23.073 to 24.164 dB when glucose levels increased, with the lowest COD of 0.7982 in comparison with the frequency. The maximum frequency shift of 0.334 GHz was generated at 300 mg/dL glucose aqueous concentration level compared to DI water, with a sensitivity of 0.254 MHz per mg/dL.

In the case of peak 2, the frequency was approximately 3.2 GHz, with both amplitude and center frequency differing when tested with samples of various concentrations (Figure 6d). The resonant frequency of the solution primarily ranged from 3.379 to 3.075 GHz for glucose concentrations varying from 25 mg/dL to 300 mg/dL with a COD of 0.98325, exhibiting a highly linear relationship. The maximum RSD of 0.71% was observed at 25 mg/dL, which indicates excellent reproducibility among all samples [16]. The absolute value of its amplitude also increased linearly from 23.452 to 33.661 dB when the glucose levels increased, with the lowest COD of 0.9617 in comparison with the frequency. The proposed device generated a maximum frequency shift of 0.883 GHz at 300 mg/dL. Additionally, this peak exhibited a sensitivity of 1.2 MHz per mg/dL. 

The peak at 3.2 GHz indicates a better correlation than that at 0.8 GHz, with the further analysis of the corresponding relationship between frequency and concentrations, matching a linear fitted curve, as depicted in Figure 6e. The calibration curves were fitted using Equation (6):

(6) y=−0.00107x+3.39102

where *y* represents the center frequency (GHz) and *x* denotes the concentrations of glucose aqueous solutions (mg/dL).

Figure 6f illustrates the summation of frequencies of peaks 1 and 2. This analysis was performed due to the higher linearity of the frequency to the magnitude and similar downtrend response to the increase in glucose concentration. The summation enhanced the detection and reduced errors, resulting in a higher sensitivity of 1.38 MHz per mg/dL and an increased correlation with improved COD of 0.9882. The calibration curves were fitted using Equation (7):

(7) y=−0.00138x+4.18714

(8) $$Glucose\;concentration_{\left(\frac{mg}{dL}\right)}=\frac{4.18714-\left(f_{r1}+f_{r2}\right)}{0.00138}$$

The response results verified that the output response of the proposed glucose biosensor ranges from 25 to 300 mg/dL with high sensitivity for resonance frequencies of 0.254 MHz at peak 1 (0.8 GHz) and 1.20 MHz at peak 2 (3.2 GHz). The summation of peaks 1 and 2 with improved COD of 0.9882 further enhanced the sensitivity up to 1.38 MHz per mg/dL. Moreover, the results of variations in serum concentration are shown in Figure S6, revealing the same trend between resonance frequency and concentration.

The glucose sensing limit of detection (*LOD*), was calculated by using the following Equation (9) as 24.59 mg/dL [29,30].

(9) LOD=3.3×SD/m

where SD is the standard deviation of the sensing response to glucose solution and m is the slope of the fitted curve. Additionally, the RSD was calculated as less than 1% (Table S1), and response time was less than 3.3 s (Table S2).

Table 2 compares the sensitivity and other performance measures of the proposed IPD biosensor with other resonator-based microwave biosensors. As indicated in the table, the proposed biosensor exhibits substantially higher sensitivity and correlation across multiple microwave biosensors.

**Table 2:** Comparison of glucose biosensor performance.

References	Structure	Sensing Method	Operation Frequency f_0_ (GHz)	Concentration Range (mg/dL)	Sensitivity (MHz per mg/dL)
[16]	LC resonator	f_r_, S_11_	0.6	30-500	1.175
[19]	SRR	f_r_	1.4	0–5000	2.60 × 10^−2^
[31]	LC resonator	f_r_, S_11_	1.4	0–72	2.6 × 10^−6^
[32]	CELC	f_r_	1.3	0–10,000	2.11 × 10^−2^
[33]	ENG unit-cell	f_r_, S_21_	1.6	2000–10,000	1.00 × 10^−2^
[34]	λ/4 resonator	f_r_, S_11_	1.68	0–30,000	1.68 × 10^−3^
[35]	CSRR	f_r_	2.45	40-140	6.3–12.5 × 10^−1^
[36]	Patch antenna	f_r_	5.0	0–250	1.09 × 10^−3^
[37]	Cavity	f_r_, S_11_	4.75	150–550	2.80 × 10^−2^
[38]	CSRR	f_r_, S_11_	2.5	0–500	0.50 × 10^−2^
Proposed biosensor	IPD LC-resonator	f_r_, S_11_	0.8, 3.2	25–300	1.38

CELC: Complementary electric-LC; SRR: Split ring resonator; ENG: Epsilon negative; CSRR: Complementary split ring resonator.

### 3.4. Microcosmic Sensing Principle and Specificity

**Figure 7:**
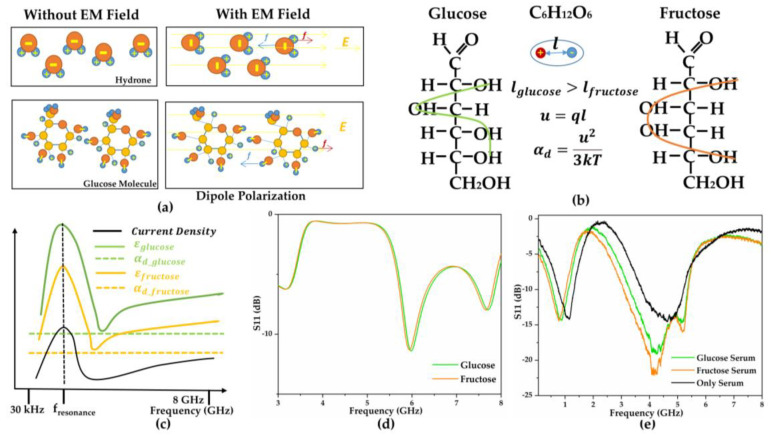
(**a**) Water and glucose molecule dipole polarization with and without the electromagnetic (EM) field. (**b**) Glucose and fructose original structure and polarizability comparison. (**c**) Glucose and fructose polarizability and permittivity response to current density. (**d**) Glucose and fructose simulation response and (**e**) measured results for 100 mg/dL glu-cose/fructose in serum compared to pure serum.

The sensing principle was developed at both microcosmic and macrocosmic levels to quantify the influence of the introduced SUT discussed in radio frequency and microwave frequency (30 kHz~30 GHz). At the microcosmic level, all particles tend to remain in dynamic equilibrium, maintaining the lowest energy state. When electromagnetic waves change the environmental conditions, the original state of molecules is disturbed through various types of polarization, leading to rearrangement. The polarization type is excited by the electromagnetic frequency; polarization density is decided by electrical field density [11,30]. Based on the detection frequency range (0.05 GHz~8.0 GHz) and liquid sensing condition, only the dipole polarization contribution to permittivity was considered, as illustrated in Figure 7a [11]. Dipole polarization performs an adaptive overall molecule rotation in the direction of the electric field. The polarization density (P) and relative permittivity ($\varepsilon^{\prime }$) can be represented by using Equations (10) and (11) [11]:

(10) $$P=\left(\varepsilon^{\prime }-1\right)\varepsilon_{0}E=\alpha_{t}E^{\prime }N^{\prime }$$

(11) $$\varepsilon^{\prime }=\frac{\alpha_{t}E^{\prime }N^{\prime }}{\varepsilon_{0}E}+1$$

where $\varepsilon^{\prime }$, $\varepsilon_{0}$ and E are macroscopic parameters: $\varepsilon^{\prime }$ represents the relative permittivity because of the polarization, $\varepsilon_{0}$ denotes the free space permittivity, E represents the existing local electrical field, and $E^{\prime }$ represents the net field as a combined effect of local field (E) and induced opposed field by induced dipoles. $\alpha_{t}$ and $N^{\prime }$ represent microscopic molecular parameters: $\alpha_{t}$ denotes the polarizability derived from the various polarizations, $N^{\prime }$ represents the number of individual dipole moments. Through this equation, the polarization density is influenced by dipole number $N^{\prime }$ and net field $E^{\prime }$ as well as the polarizability $\alpha_{t}$. Additionally, the relative permittivity derived from the dipole number $N^{\prime }$ contributes to total polarization and polarizability. 

Herein, our sensing sample materials were composed of water and glucose molecules with frequencies at 0.8 GHz and 3.2 GHz; the polarization ($\alpha_{t}$) mainly consisted of the dipole polarization ($\alpha_{d}$), following Equations (12) and (13) [30]:

(12) u=ql

(13) $$\alpha_{d}=\frac{u^{2}}{3kT},\;uE\;\ll kT$$

where u is the permanent dipole moment depending on dipole type, l is the distance between +q and −q shown in Figure 7b. Hence, the permittivity depends on the field density when sensing the fixed SUT in a frequency-varying electromagnetic field. When sensing in a fixed field (E), the permittivity depends on the dipole type, internal structure, and number. We considered glucose and fructose as a comparison group; glucose appears more polar because of its asymmetric construction, contributing to higher polarizability and sensitivity permittivity variation to frequency [30]. 

As a result, Figure 7c illustrates the average current density responding to the frequency, corresponding polarizability, and permittivity variation. In addition, we simulated and measured the 100 mg/dL glucose and fructose serum solutions and their various responses, demonstrating the specificity of the proposed sensor, as depicted in Figure 7d,e. Herein, the resonance occurring in the equilibrium condition represented the maximum electromagnetic transmission at that frequency with the slightest loss [39]. Furthermore, the amplitude of the electromagnetic oscillation attained its peak, which was reflected in the peak of S11.

Based on the above theory, electromagnetic sensing provides the ability to determine various materials even with the same chemical formula because of the different frequency responses that derive from their internal structures and multiple concentrations due to diverse responses derived from the dipole number. From this perspective, there are two optimization methods for improving selectivity: increasing the surface current density to enhance the response difference, or assisting chemical bonding to emphasize the material. We investigated the latter approach through the utilization of GOx and various combination methods as shown in Figures S3 and S4, demonstrating the specific chemical combination effective in strengthening response specificity. 

### 3.5. Macroscopic Sensing Analysis

At a macroscopic level, also viewed as the device level, the dropped glucose analyte is regarded as a new filling medium with enhanced permittivity. Based on the equivalent circuit model illustrated in Figure 1b and calculations of Equations (1) and (4), the increase in total capacitance decreases the resonant frequency, which primarily relies on glucose permittivity. The ideal complex permittivity of glucose solutions, composed of real and imaginary parts, can be extracted from the Debye relaxation model and represented using Equation (14) [40]:

(14) $$\varepsilon_{g}=\varepsilon_{real}-j\varepsilon_{imag}=\left[\frac{\left(\varepsilon_{s}-\varepsilon_{\infty }\right)}{1+\omega^{2}\tau^{2}}+\varepsilon_{\infty }\right]+j\left[\frac{(\varepsilon_{s}-\varepsilon_{\infty })\omega \tau }{1+\omega^{2}\tau^{2}}\right]=\varepsilon_{\infty }+\overset{p}{\underset{k=1}{\sum }}\frac{\Delta_{\varepsilon k}}{1+j\omega \tau_{k}}$$

where ω represents the angular frequency, ω=2πf; p denotes the fitting order of the Debye model, p=1; $\varepsilon_{\infty }$ indicates the relative permittivity at infinite frequency; and $\Delta_{\varepsilon k}$ and $\tau_{k}$ denote the dispersion values and relaxation time, respectively. The aforementioned values are estimated using the fitted curve based on the measured values, as indicated in Equations (15)–(17) [40]:

(15) $$\varepsilon_{\infty }=c_{glucose}^{2}\times 1.073\times 10^{-6}+c_{g}\times 2.29\times 10^{-3}+9.824$$

(16) $$\Delta_{\varepsilon k}=c_{glucose}^{2}\times 0.916\times 10^{-6}-c_{g}\times 2.29\times 10^{-3}+69.28$$

(17) $$\tau_{k}=c_{glucose}^{2}\times 0.2156\times 10^{-7}-c_{g}\times 0.276\times 10^{-3}+9.21$$

where $c_{glucose}$ represents the glucose concentration (mg/dL). The calculated permittivity values of various glucose concentrations were approximately 78, which also could be extracted by artificial neural networks [41].

**Figure 8:**
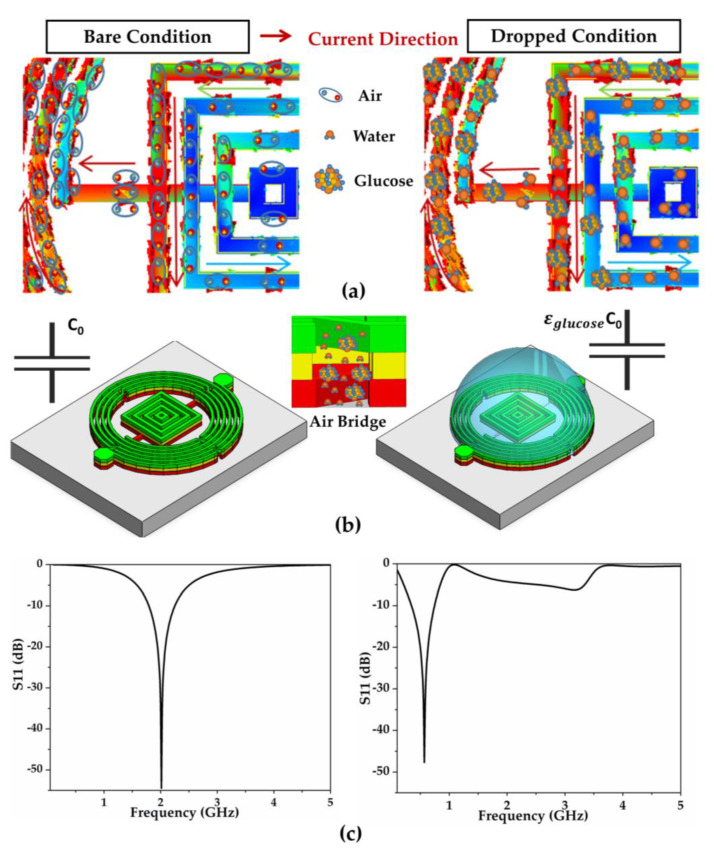
Comparison of bare and dropped condition. (**a**) Molecular polarization condition corresponding to current di-rection. (**b**) Corresponding equivalent capacitor. (**c**) Resonance simulation corresponding to without and with SUT.

Figure 8a depicts the surface molecule distribution corresponding to surface current density, while Figure 8b,c compares the analysis and simulation of unloaded and dropped conditions equivalently. Considering the dropped analyte as the filling medium, the value of medium permittivity ($\varepsilon_{0}$) at 1 increased to several dozens ($\varepsilon_{glucose}$) based on Equation (4). Additionally, the air-bridge capacitance $C_{0}$ increased to $\varepsilon_{glucose}C_{0}$ and contributed to the overall increase in the device capacitance, which decreased the resonant frequency to the corresponding value based on Equation (1). The resonant frequency exhibited an evident decline from 2 GHz to 0.571 GHz through a simulation based on the change in filling medium permittivity from 1 to 78. 

Furthermore, considering the permittivity change principle, the frequency detection resolution (FDR) was defined and represented as the ratio of resonant frequency to the variation in dielectric constant derived from concentrations, as indicated in Equations (18) and (19) [42]:

(18) $$\Delta f_{r}=f_{r}\left(loaded\right)-f_{r}\left(unloaded\right)$$

(19) $$FDR=\frac{\Delta f_{r}}{\Delta_{\varepsilon r}}$$

where $\Delta_{\varepsilon r}$ represents the change in permittivity of the sample. The relationship between the frequency shift and exported glucose permittivity could be calculated using Equations (15)–(17). As the proposed biosensor exhibits an FDR of 464 MHz for each unit permittivity variation, it can be used as a candidate to detect other aqueous samples with various values of permittivity. 

## 4. Conclusions

To identify the glucose level in an aqueous solution, we propose a reusable microwave resonator-based biosensor fabricated using IPD technology. The proposed biosensor detected glucose at concentrations ranging from 25 to 300 mg/dL, with a sensitivity of 1.20 MHz per mg/dL, and showing an LOD of 24.59 mg/dL. Additionally, an optimization method for analyzing the summation of two peak frequencies improved the linear relationship corresponding to various glucose concentrations with a sensitivity of 1.38 MHz per mg/dL. Thus, the sensitivity of the biosensor was substantially higher than those of existing biosensors. Further considerations for the sensing microcosmic principle and the macroscopic analysis based on the RLC circuit were discussed. Moreover, the periodic detection and morphological analysis showed that no frequency deterioration and chemical binding occurred during the entire experiment, thus validating the reusability and reliability of the biosensor for real-time glucose detection. The permittivity response was investigated with a sensitivity of 466 MHz for unit permittivity variation, implying the proposed biosensor can potentially detect biological aqueous solutions with various permittivity differences. Meanwhile, the GOx-assisted approach increased the biosensor’s selectivity to glucose through the specific reaction between GOx and glucose, proving its potential in clinical application.

## Figures and Tables

**Table 1:** Coefficients for various shapes.

**Layout**	$c_{1}$	$c_{2}$	$c_{3}$	$c_{4}$
Square	1.27	2.07	0.18	0.13
Hexagonal	1.09	2.23	0.00	0.17
Octagonal	1.07	2.29	0.00	0.19
Circle	1.00	2.46	0.00	0.20

## Data Availability

Not applicable.

## Supplementary Materials

The following are available online at https://www.mdpi.com/article/10.3390/bios11120508/s1, Figure S1: Unpackaged chip. (a) Front side with Au wire bonding connecting to 50-ohm matching line; (b) backside with via holes. Figure S2: Different liquid volume response curves. Figure S3: The variations in response of 100 mg/dL glucose and fructose dissolved in serum with/without glucose oxidase. Figure S4: Different mixture response curves. Figure S5: Reaction principle. Figure S6: Response curves for different glucose concentrations in serum. Table S1: RSD calculation of the peak 2 frequency. Table S2: 8719E full frequency band sweep time (0.05 to 13.5 GHz ms).
