# Supplementary material for: Permittivity-Inspired Microwave Resonator-Based Biosensor Based on Integrated Passive Device Technology for Glucose Identification
Source: Biosensors (Basel). 2021 Dec 9;11(12):508. doi: 10.3390/bios11120508 (PMC8699625; doi:10.3390/bios11120508)
Supplement: Supplementary file 1 [file biosensors-11-00508-s001.zip › biosensors-1438358-supplementary.pdf]

biosensors

Supplementary Materials

Permittivity-inspired Microwave Resonator-Based Biosensor Based on Integrated Passive Device Technology for Glucose Identification

Wei Yue [1], Eun-Seong Kim [1], Bao-Hua Zhu [1], Jian Chen [1], Jun-Ge Liang [2,*] and Nam-Young Kim [1,*]

1 Radio Frequency Integrated Circuit (RFIC), Kwangwoon University, Kwangwoon-ro, Nowon-gu, Seoul 01897, Korea; yuewei@kw.ac.kr (W.Y.); esk@kw.ac.kr (B.-H.Z.); cnjacob@kw.ac.kr (J.C.);
2 Engineering Research Center of IoT Technology Applications (Ministry of Education), Department of Electronic Engineering, Jiangnan University, Wuxi 214122, China
* Correspondence: zhuwangwhy@kw.ac.kr (E.-S.K.); jgliang@jiangnan.edu.cn (J.-G.L.); nykim@kw.ac.kr (N.-Y.K.)

1. Front and backside of the unpackaged chip

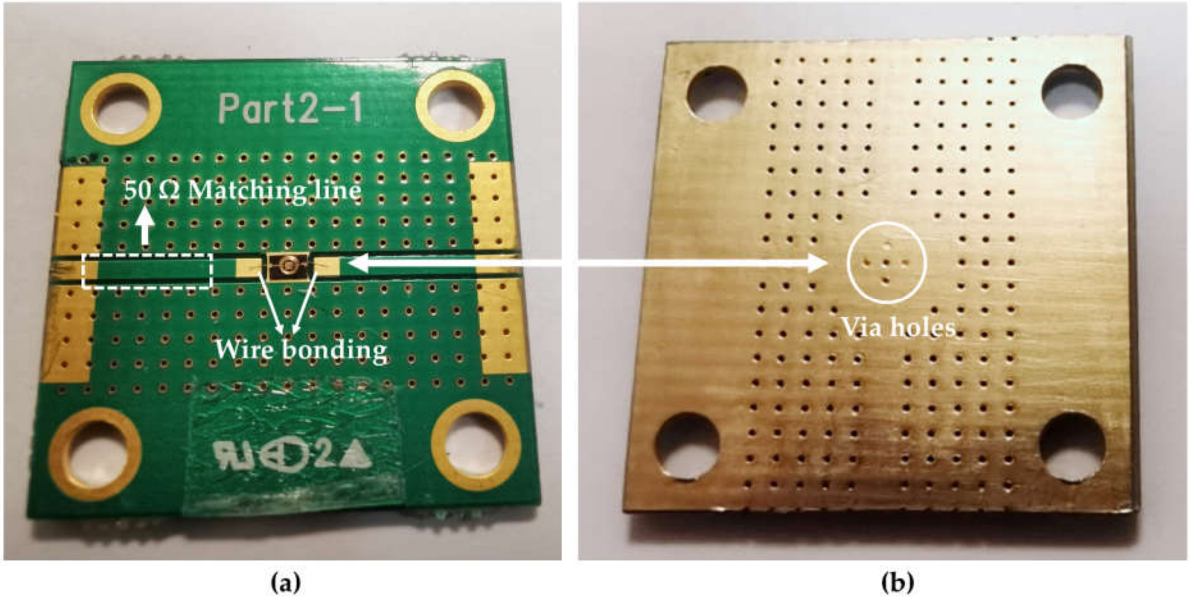

Figure S1. Unpackaged chip. (a) Front side with Au wire bonding connecting to 50-ohm matching line; (b) backside with via holes.

2. Liquid Volume Response

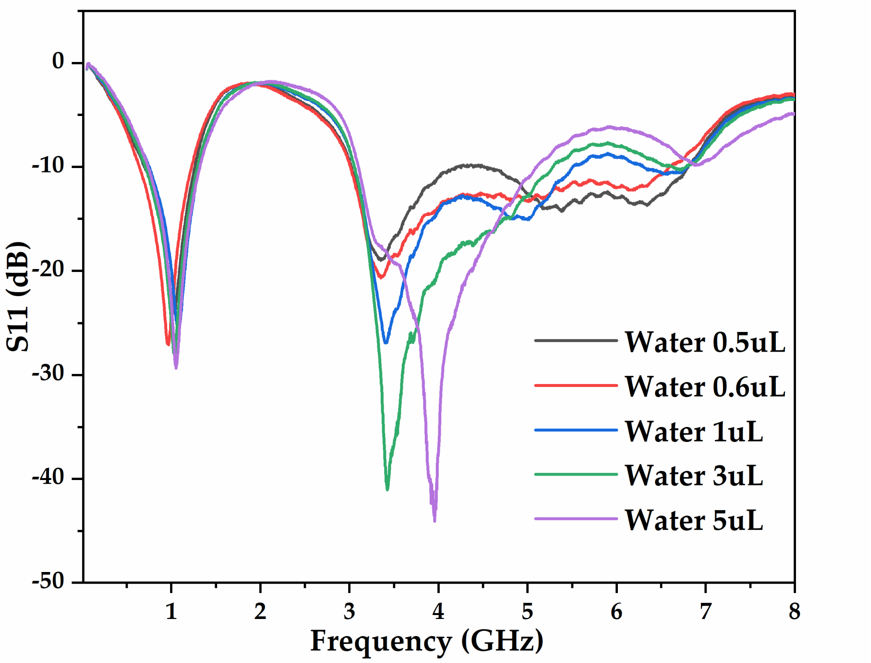

Figure 2. Different liquid volume response curves.

We consider a droplet as a capacitor influenced by volume and amounts, which appears as a capacitor-like effect. After introducing the droplet, the internal device capacitor and the introduced capacitor lead to a resonance frequency shift. The first peak was considered the internal capacitor increase; the second peak was the droplet capacitor introduction, influenced by the liquid type and volume [1].

In Figure S1, based on the above principle, first, the size of the bare chip area is 980 μm × 800 μm; at a low volume such as 0.5 μL, the volume is enough to cover the chip area, supporting changes in environmental permittivity. In contrast, the second peak is not apparent and sharp. As the liquid volume increases, the first peak appears as a minor shift, and the second peak changes more obviously with a larger amplitude (better transmission), proving the assumption is correct.

Additionally, we consider that the droplet shape strongly influences the results; 5 μL is better controlled at a fixed shape at the fixed groove. Therefore, as long as the volume is determined and set up at a high value, the glucose solution concentration contributes to the first and second peaks.

3. Standard Deviation (SD) and Relative Standard Deviation (RSD) Calculation

Table S1. RSD calculation of the peak 2 frequency.

Concentration	1	2	3	Average value	SD	RSD
25 mg/dL	3.35720	3.36814	3.41289	3.37941	0.02409	0.7128%
50 mg/dL	3.35720	3.35124	3.34129	3.34991	0.00656	0.1958%
75 mg/dL	3.30052	3.29555	3.28263	3.29290	0.00754	0.2290%
100 mg/dL	3.28263	3.28263	3.27666	3.28064	0.00281	0.0857%
125 mg/dL	3.26771	3.24186	3.23191	3.24716	0.01509	0.4647%
150 mg/dL	3.23191	3.23191	3.22794	3.23059	0.00187	0.0579%
175 mg/dL	3.21799	3.21103	3.22197	3.21700	0.00452	0.1405%

200 mg/dL	3.17071	3.14885	3.13295	3.15083	0.01548	0.4913%
225 mg/dL	3.10364	3.14839	3.14839	3.13347	0.02109	0.6731%
250 mg/dL	3.11856	3.11856	3.10762	3.11491	0.00516	0.1657%
275 mg/dL	3.07680	3.10265	3.07680	3.08541	0.01219	0.3951%
300 mg/dL	3.08276	3.07680	3.06586	3.07514	0.00700	0.2276%

*1,2,3 are experiment times; SD: standard deviation; RSD: relative standard deviation.

$$SD(Average) = 0.010283, m = 0.00138$$

$$LOD = 3.3 * SD/m = 24.59058\ mg/dL$$

4. Glucose/Fructose Serum Solution Reaction with Glucose Oxidase (GOx)

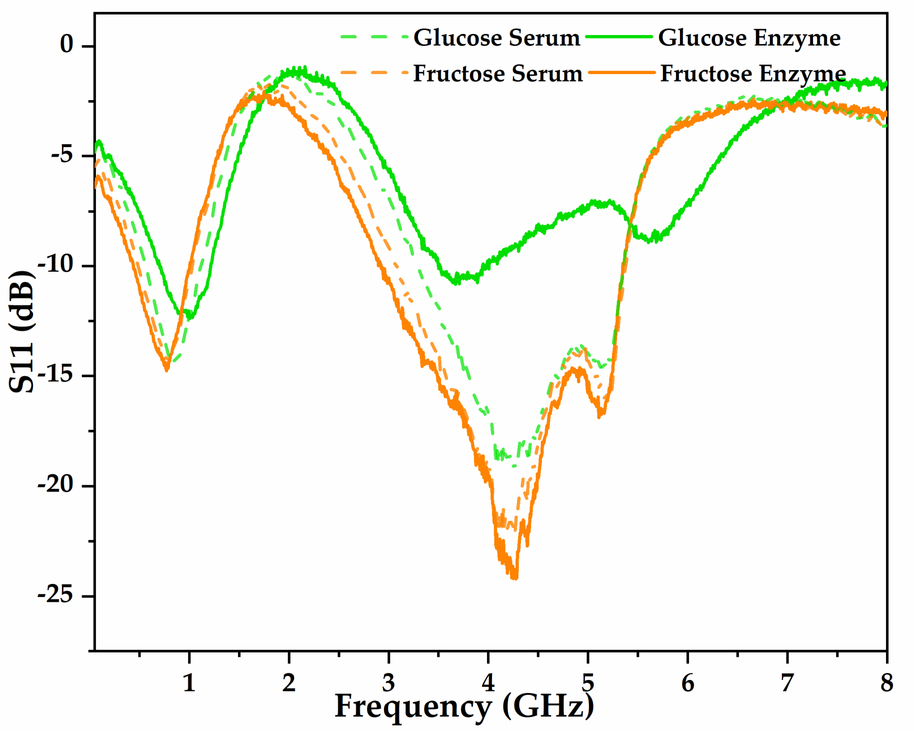

Figure 3. The variations in response of 100 mg/dL glucose and fructose dissolved in serum with/without glucose oxidase.

Figure S3 depicts the variations in response of 100 mg/dL glucose and fructose serum solution after mixing and reacting with GOx solution. Before adding GOx solution, there was a minor distinction between glucose and fructose. The GOx chemical reaction with glucose enhanced the difference (Figure S3 and Figure S4), showing an obvious variation in glucose serum solution response, whereas the fructose solution shifted only a little [2]. The results proved that the mixture detection approach could avoid the effect of impurities and enhance the specificity of the proposed biosensor.

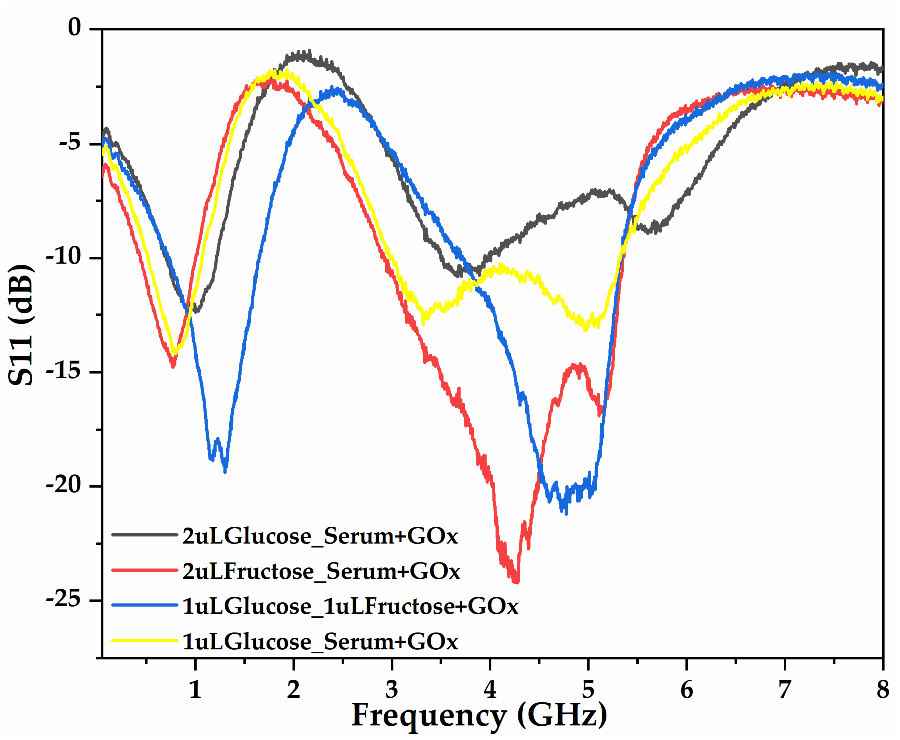

Figure S4. Different mixture response curves.

In Figure S4, the black line represents the mixture composed of 2 μL 100 mg/dL glucose serum solution with 3 μL GOx solution (16.80 units); red line represents the mixture composed of 2 μL 100 mg/dL fructose serum solution with 3 μL GOx solution (16.80 units); blue line depicts the mixture composed of 1 μL 100 mg/dL fructose and 1 μL 100 mg/dL glucose serum solution with 3 μL GOx solution (16.80 units); and yellow line depicts the mixture composed of 1 μL 200 mg/dL glucose serum solution with 4 μL GOx solution (22.40 units).

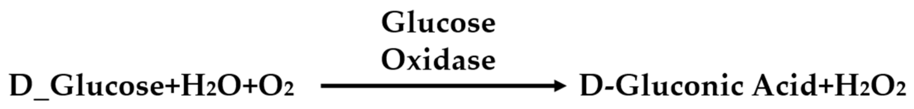

Figure S5. Reaction principle.

One unit will oxidize 1.0 μmole of β-D-glucose to D-gluconolactone and H2O2 per min at pH 5.1 at 35 °C, equivalent to an O2 uptake of 22.4 μl per min. If the reaction mixture is saturated with oxygen, the activity may increase by up to 100% [3].

5. Glucose Serum Solution Response

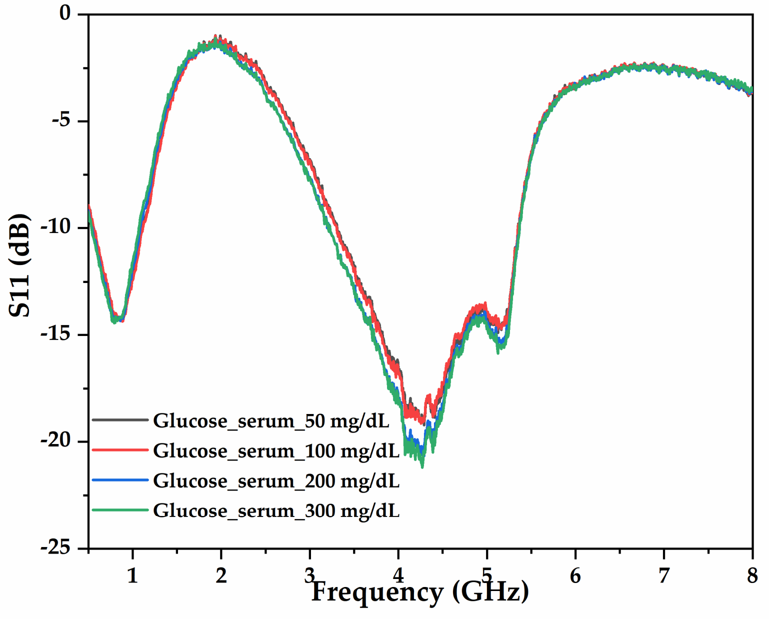

Figure S6. Response curves for different glucose concentrations in serum.

6. Response Time Analysis

Table S2. 8719E full frequency band sweep time (0.05 to 13.5 GHz ms).

Measurement	51	201	401	1601
Uncorrected	484 /597	553 /1014	614/1490	926/4336
One-port calibration	484 /597	553 /1014	614/1490	926/4336
Two-port calibration	996/1222	1133/2069	1259/3057	1876/8892

For the response time of our proposed microwave biosensor, the dynamic response was exported by the stable time based on the sweeping counts and single time. Herein, we used VNA 8719ES ranging from 0.05 GHz to 8 GHz, and the measurement needed no more than 3 sweeps to be stable.

The calculation is

$$t_{single} = \frac{8 - 0.05}{13.5 - 0.05} \times 1876 \approx 1109\, ms \quad (1)$$

$$t_{total} = 3 \times t_{single} \approx 3327\, ms = 3.3\, s \quad (2)$$

By the calculation, our response time is less than 3.3 s, which could be further reduced if we reduced the number of detection points.

References

1. Bisigato, A.; Hardtke, L.; Del Valle, H. Soil as a capacitor: Considering soil water content improves temporal models of productivity. *J. Arid Environ.* **2013**, *98*, 88–92, doi:10.1016/j.jaridenv.2013.08.004.

2. Yoo, E.-H.; Lee, S.-Y. Glucose biosensors: an overview of use in clinical practice. *Sensors (Basel).* **2010**, *10*, 4558–4576, doi:10.3390/s100504558.

3. https://www.sigmaaldrich.com/KR/en/product/sigma/g7141?context=product.
